# THE USE OF ARCHIVED GIEMSA-STAINED BLOOD SMEARS AND RDT FOR PCR-BASED GENOTYPING OF *Plasmodium* v*ivax* MEROZOITE SURFACE PROTEIN-1 IN CENTRAL KALIMANTAN PROVINCE, INDONESIA

**DOI:** 10.21010/Ajid.v16i1.3

**Published:** 2021-12-21

**Authors:** Trilianty Lestarisa, Heny Arwati, Yoes Prijatna Dachlan, Soedjajadi Keman, Din Safruddin

**Affiliations:** 1 Doctoral Program on Public Health, Faculty of Public Health, Universitas Airlangga, Surabaya, Indonesia; 2 Department of Public Health, Faculty of Medicine, Universitas Palangka Raya, Palangka Raya City, Indonesia; 3 Department of Medical Parasitology, Faculty of Medicine, Universitas Airlangga, Surabaya, Indonesia; 4 Department of Environmental Health, Faculty of Public Health, Universitas Airlangga, Surabaya, Indonesia; 5 Eijkman Institute for Molecular Biology, Jakarta, Indonesia; 6 Department of Parasitology, Faculty of Medicine, Hasanuddin University, Makassar, Indonesia

**Keywords:** Malaria, Pvmsp-1, indigenous and migrant population, Central Kalimantan Province of Indonesia

## Abstract

**Background:**

*Plasmodium vivax* is transmitted most across the country of Indonesia. The country has set out a malaria elimination program by 2030. The information on genetic diversity of malarial parasites relates to malaria transmission in an endemic area may provide the information that can help the malaria control program to achieve the target. Therefore, the purpose of this study was to determine the genetic diversity of the Pvmsp-1 gene in Central Kalimantan Province.

**Materials and Methods:**

Samples were 140 of archived Giemsa-stained blood smear and rapid detection test. Samples were divided into the indigenous and migrant populations. After confirmation by single-step PCR, only *P. vivax* and mixed infection samples were amplified to nested PCR for genotyping of Pvmsp-1 allelic variation in segments F1, F2, and F3.

**Results:**

Genotyping of 23 PCR positive samples resulted in 13 genotypes. In segment F1, three allelic variants type A containing subtype A1 (1,050 bp), A2 (350 bp), A3 (150 bp), and type B (100 bp). In segment F2, mono genotypes were detected as variant type A (1,050 bp) and type B3 (150 bp), multiple genotypes were detected as type B containing subtype B1 (250 bp), B2 (200 bp), and B3 (150bp). In segment F3, three allelic variants generated from four mono genotypes were type A (350 bp), type B (300 bp), and two type C (250 bp).

**Conclusion:**

The low allelic variation of Pvmsp-1 gene may reflect the actual situation of the low malaria endemic status of the study sites.

## Introduction

Malaria in Indonesia remains an important health burden, and 10.4% was contributed to the malaria cases worldwide, and as second contributor after India (WHO, 2020). However, there was a declined of malaria cases in Indonesia from 465,700 in 2010 to 235,700 in 2020, followed by the decreased of national annual parasite incidence (API) from 1.96 in 2010 to 0.87 in 2020 (Kemenkes, 2021). Indonesia has set out a plan to eliminate malaria in the whole country by 2030, with phased target by region depending upon malaria endemicity (MOH, 2020). To achieve the target of malaria elimination, the movement of individuals from malaria endemic regions to non-endemic areas must be controlled to limit reintroduction of malaria to non-endemic areas but also to limit global genetic diversity and its spread in various areas (Arnott et al., 2012).

Central Kalimantan is one of the developing provinces in Indonesia with the majority of the population working in the agriculture, plantation and mining sectors. This has led to a large number of new land clearing for oil palm plantations and the increase in traditional gold mining practices, which is one of the reasons this province has not succeeded in eliminating malaria incidence (BPS Prov. Kalteng, 2018). In 2019, API in this province was 0.1. Three out of 14 districts are now still doing efforts to achieve malaria elimination, such as Kapuas, Gunung Mas, and Murung Raya Districts where the endemicity is low (Kemenkes, 2021). Palangka Raya City, the capital of this province has been certified to be free of malaria (Pemko Palangka Raya, 2018). The indigenous people of Kalimantan (Borneo) Island is called Dayak (Sada et al., 2019). The illegal gold mining attracted migrants to come to the island for settling. The migrant people came from outside the districts even from outside the island. The migrant people was categorized as non Dayak people who have resided in the study area for more than three months. The migrant people come from different ethnicities and origin of stay possibly have different immune status against malaria and bring in malaria parasite that ultimately cause genetic diversity of parasites (Rodriguez et al., 2006). Human migration is a major factor in the reemergence and spread of malaria that transmitted to the local population (Arnott et al., 2012). Assessing malaria parasites genetic diversity may be useful to elucidate the transmission dynamic and re-introduction of malaria in the area (Nabet et al., 2016).

*Plasmodium vivax* infection is transmitted across most the Indonesian archipelago (Elyazar et al., 2012). During 2019, the *P. vivax* cases was estimated to be lower (37.19%) than *Plasmodium falciparum* (60.88%) (WHO, 2020). *P. vivax* is one of the human malaria parasite species causing benign malaria (Imwong et al., 2005). The vivax cases can lead into debilitating illness (Imwong et al., 2005), and widely distributed (Abdullah et al., 2013), and more difficult to control and eliminate than *P. falciparum* in areas where they are sympatric (Jennison et al., 2015). Hypnozoite is dormant stage of *P. vivax* can cause relapse in weeks or months after the initial infection, complicate the disease identification either as re-infection or recurrence, and cause possible failure of the treatment (Thanapongpichat et al., 2019).Unlike *P*. *falciparum*, the in vitro continuous culture of *P. vivax* is still difficult to establish (Bermudez et al., 2018). Blood from *P. vivax*-infected patients is the only source of samples for molecular studies (Thanapongpichat et al., 2019). Currently, during the Covid-19 pandemic, malaria remains a health problem worldwide and affects various aspects of life. The pandemic has caused a problem in malaria field survey especially in collecting the fresh blood samples from patients. Alternatively, the archived Giemsa-stained blood smears (GSBS) and rapid diagnostic tests (RDT) were used to isolate the parasites DNA to achieve this purpose.

Merozoite surface protein-1 is a protein found on the surface of merozoites which is important for parasite invasion into erythrocytes and is a potential vaccine candidate (Beeson et al., 2016). Investigating the genetic diversity of *P. vivax* populations is essential that may contribute to malaria control and elimination. *Plasmodium vivax* merozoite surface protein-1 gene (Pvmsp-1) is the genetic marker that most commonly used to determine the genetic diversity of *P. vivax*. The Pvmsp-1 gene contains 13 alleles conserve and highly variable blocks. The variable blocks are containing block 2 (F1 region), 6-8 (F2 region), and 10 (F3 region) (Imwong et al., 2005). Analysis of Pvmsp-1 for F1, F2 and F3 segments allelic variation using polymerase chain reaction and restriction fragment length polymorphism (PCR-RFLP) in Thailand has been reported to have 5, 2, and 3 allelic variants, respectively (Raza et al., 2013), in Pakistan were 23, 41 and 23 allelic variants, respectively (Raza et al., 2013), and in India were 6, 2, and 3 variants, respectively (Kibria et al.,2015).

The genetic population structure of *P. vivax* in Central Kalimantan Province, Indonesia has not been systematically studied. Investigations on the Pvmsp-1 allelic variation in Indonesia are very limited. Therefore, this study aims to determine the PCR-based allelic variation of the Pvmsp-1 from indigenous and migrant populations in Central Kalimantan Province as background information to guide in understanding the transmission of *P. vivax* infection in these areas.

## Materials and Methods

### Sample Collection and Ethics

Samples used in this study were archived Giemsa-stained thick and thin blood smears (GSBS) and rapid detection test (RDT) cartridges collected during 2017 to 2020 from public health service (Puskesmas) of two districts, Kapuas District and Gunung Mas District, where malaria is still endemic and one municiplity (Palangka Raya) where malaria is eliminated. The samples were then differentiated into samples from indigenous and migrant people based on the data from the health centers. The blood films have been examined to be positive for malaria parasite by two certified malaria microscopists. The specimens were transferred to our laboratory by packages delivery service in 2021. The study has been approved by the Ethical Review Committee of Faculty of Dental Medicine Universitas Airlangga, Surabaya, Indonesia as stated on the certificate number 008/HRECC.FODM/I/2021.

### DNA extraction

In order to perform PCR amplification, the DNA was extracted from archived GSBS and used RDT cartridges. Each GSBS were scratched off by using a sterile scalpel. Each individual RDT cartridges was opened using scissors and forceps and the nitrocellulose strip was removed from the plastic case, then any plastic covering on the strip was stripped off. The proximal third of the test strip containing DNA was cut and subsequently used for DNA extraction as described elsewhere (WWARN, 2014). The collected materials from blood smears and RDTs were then transferred to a sterile tube. The DNA extraction was done using QIAamp DNA Blood Mini Kit (Qiagen, Hilden, Germany) following manufacturer’s instruction.

### Molecular confirmation of *Plasmodium* species

The DNA was then amplified for single step PCR using primers and PCR condition based on previously described (Patsoula et al., 2003; Arwati et al., 2013) for the confirmation of species of *Plasmodium*. Primers used in this PCR were based on the sequences of small-sub unit ribosomal RNA (ssu-rRNA) of *P. falciparum* and *P. vivax*. This PCR system used 3 primers in one tube which amplified sequence common to rRNA of both *Plasmodium*, and specific for each *P. vivax* and specific for *P. falciparum*. The three primers produced a 266 bp product specific for *P. vivax*, and a 346 bp product specific for *P. falciparum*. The PCR products were then verified through electrophoresis on 2% agarose gel and visualized on the UV light and documented. The positive samples determined by PCR were then used for nested PCR amplification to analyze the allelic variation of Pvmsp-1.

### Amplification of PvMSP1 gene

The three polymorphic regions of PvMSP-1 gene, namely F1, F2 and F3 were analyzed according to the previously described method with slight modification (Imwong et al., 2005; Suphakhonchuwong et al., 2018). Total volume of PCR mixture was 20 µl, containing 12.5 µl of 2x PCR mixture (Intron, Singapore), 5 µl of DNA template and 20 pmol of each primer. Primers and PCR condition were as described (Imwong et al., 2005) with slight modification. PCR products were directly analyzed using electrophoreses in agarose gels. Five microliters of PCR product were mixed with 1µl of loading buffer and applied to 2% agarose gel. The fragments of DNA were visualized by UV transillumination after ethidium bromide staining. The size of amplified fragments was compared by size to a 100 bp ladder marker (Invitrogen, 100 bp).

### Data analysis

The frequency of each Pvmsp-1 allele was calculated based on the presentation of samples containing that allele from the total sample in each population. Allelic variation was determined based on the number and the size of PCR fragment of each allele in each sample. The Multiplicity of Infection (MOI) was defined as the number of parasitic genotypes per infection and was obtained by dividing the total number of fragments by the number of positive isolates containing allele in the same loci. Isolates with more than one genotype were considered as a polyclonal infection while the presence of a single allele was considered as monoclonal infection.

## Results

### Number of samples

A total of 140 samples consisted of 75 GSBS, and 65 RDT cartridges. Based on the data obtained from health centers, the samples were then differentiated into 85 samples of indigenous people, and 55 samples were from migrant people. The basic characteristic of the samples is shown in Table 1.

**Table 1:** Microscopy and RDT identification of species of *Plasmodium* among indigenous and migrant population in Central Kalimantan Province during 2017-2020

Species	Indigenous	Migrant	Total
*P.falciparum*	35 (41.18)	26 (47.27)	61 (43.57)
*P.vivax*	48 (56.47)	27 (49.09)	75 (53.57)
Mix	0 (0)	1 (1.82)	1 (0.71)
Negative	2 (2.35)	1 (1.82)	3 (2.14)

Total	85 (100)	55 (100)	140 (100)

### Baseline demographic data

The archived GSBS and RDT samples were collected from patients mainly aged 15-65 years old, male patients was the most found, and gold miner was the most occupation among the patients in both population (Table 2).

**Table 2:** Baseline demographic data of the patients

	Community	

	Indigenous (%) n=85	Migrant (%) n=55
**Age (year)**		
15-35	65 (76.47)	44 (80)
36-65	20 (23.53)	11 (20)

**Gender**		
Male	78 (91.8)	51 (92.7)
Female	7 (8.2%)	4 (7.3)

**Occupation**		
Gold miner	59 (69.4)	47 (85.5)
House wife	7 (8.2)	4 (7.3)
Private-employed	11 (13)	3 (5.4)
Civil servant	4 (4.7)	1 (1.8)
Army/Police	4 (4.7)	0 (0.0)

### Molecular confirmation of *Plasmodium* species

Thirty-five (25%) out of 140 samples were detected positive by single step PCR, consisted of 25 samples (71.43%) of microscopic origin, and 10 samples (28.58) of RDT origin. False negative by this PCR was 105 samples, consisted of 50 samples (47.62%) from microscopy samples, and 55 (52.38%) from RDT samples. Eleven samples (18.03%) out of 61 samples of *P. falciparum* microscopic samples were identified as same species. From 64 *P. vivax* microscopic samples only one sample (1.35%) were identified as *P. falciparum*, 3 (4.05%) were identified as *P*. vivax, and 19 (25.68%) were mix infection of both species. Only samples positive *P. vivax* and mix infection were used for nested PCR. One sample detected as *P. malariae* by microscopic examination, but negative by PCR. There was no false positive in this PCR. The results of single step PCR were presented in Table 3.

**Table 3:** Number (%) of *Plasmodium* species identified by microscopy examination and RDT compared with that of single step PCR.

Microscopy and RDT		Single Step PCR (%)				

		Pf	Pv	Pm	Mix	Neg
Pf	61	11(18.03)				50(81.97)
Pv	74	1(1.35)	3(4.05)		19(25.68)	51(68.92)
Pm	1					1(100)
Mix	1				1(100)	
Neg	3					3(100)

Total	140	140				

### Allelic variation of Pvmsp-1 and MOI

**Figure 1:**
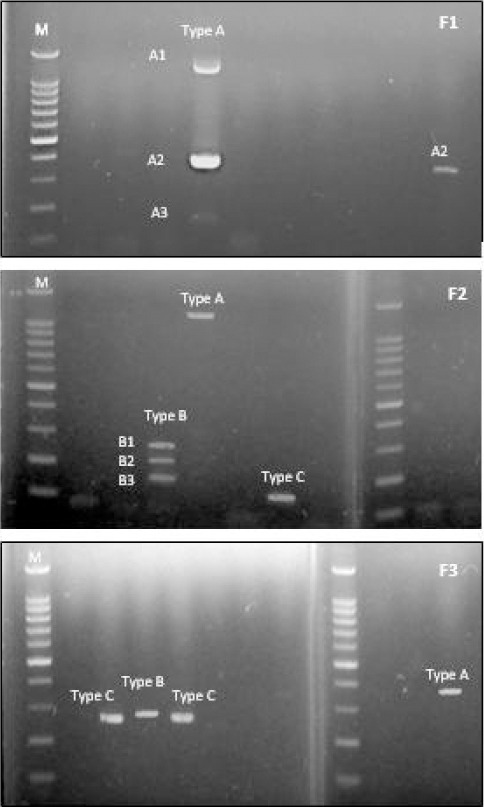
Allelic variation of Pvmsp-1 gene using nested PCR. Only small number of sample contains allele F1, F2, and F3 as well as the variation of number and size of PCR fragment.

Polymorphism of segment F1, F2, and F3 of Pvmsp-1 gene was performed in 23 samples, consisted of three samples of *P. vivax* and 20 of mix infection, 13 samples among them were from indigenous, and 10 samples were from migrant population. Only 6 samples (26.09%) out of 23 were detected containing Pvmsp-1 gene, where each three of which were from indigenous and migrant population, respectively. Genotyping based on the size of PCR fragments in segment F1, F2 and F3 within small number of isolates 13 genotypes were observed in both indigenous and migrant populations. In segment F1, three allelic variants generated from one multiple genotypes, and two mono genotypes. The multiple genotypes were determined as variant type A containing subtype A1 (1,050 bp), A2 (350 bp), A3 (150 bp). Mono genotypes were detected as type B (350 bp). In segment F2, three allelic variants were generated from two mono genotype (type A and type C), and one multiple genotype (type B). Mono genotypes were detected as variant type A (1,050 bp) and type C (150 bp). Multiple genotypes were detected as type B containing sub type B1 (250 bp), B2 (200 bp), and B3 (150 bp). In segment F3, three allelic variants generated from four mono genotypes were type A (350 bp), type B (300 bp), and two type C (250 bp). The detail results are summarized in Table 3 and visualized in Figure 1. Based on the data in Table 3, MOI within the indigenous population was 1.67, and within the migrant population was 1.0.

**Table 4:** Summary of allelic variation of Pvmsp-1 gene among indigenous and migrant population in Central Kalimantan Province.

	Indigenous			Migrant		

	Type and size of fragment (bp)	Number of genotypes	Frequency (%) n=13	Size of fragment (bp)	Number of genotypes	Frequency (%) n=10
F1	Type A Sub type A1: 1,050 A2: 350 A3: 150	3	1 (7.69)	Type B: 350	1	1 (0.10)

F2	Type A: 1,050 Type B: Sub type B1: 250 B2: 200 B3:150	3	1 (7.69)	Type C: 100	1	1 (0.10)

F3	Type B: 300	1	1 (7.69)	Type A: 350	1	1 (0.10)
	Type C: 250	1	1 (7.69)	Type C: 250	1	1 (0.10)

Total		10	4		4	4

## Discussion

This study is the first report on the allelic diversity of Pvmsp-1 gene among the samples collected from the indigenous and migrant population of Central Kalimantan Province, Indonesia. Most male adult patients related to gold miner as the most occupation in studied areas. All miners were male aged between 15-65 years old. The gold miners are the most popular contributors to the total number of malarial cases in this study, where in indigenous population was 59 (42.14%), and migrant population was 47 (33.57%). Illegal gold mining mostly are located on the slopes of mountain. The difficult access to reach it may contributes to the increased of malaria cases due to the increasing contact with *Anopheles* mosquito during the journey to and from gold mining (Indriyati et al, 2018). Based on some information collected from various local newspapers and news web pages, illegal gold mining which creates many deserted digging holes has become a major concern for the Central Kalimantan Provincial Government because it causes environmental damage and becomes a breeding site for the *Anopheles* mosquito.

During the Covid-19 pandemic, many health services and program were substantially impacted including malaria active and passive cases detection and surveillance. The archived GSBS and RDTs PCR analysis apparently provide a reliable, alternative tool to evaluate the ongoing malaria endemic situation as well as the malaria transmission dynamics in the study sites (Cnops et al, 2010; Nguyen et al, 2019). The results of molecular identification of *Plasmodium* clearly indicate that, DNA could be isolated from GSBS and RDT using the described method and that PCR system using this DNA worked well for the detection and genotyping of *Plasmodium* species. Mixed infection was more detectable by single step PCR (20 samples or 14.29%) than both microscopy and RDT. Misdiagnosis on microscopy is often associated with the reading proficiency of the microscopist and the blood film preparation although the finding of parasite on Giemsa-stained blood smear is a gold standard of malaria diagnosis. Although RDT is also recommended by WHO for rapid use in the diagnosis of malaria in certain circumstances, the false negative on RDT due to the improper use of RDT, storage and parasite density may occur.

Genotyping parasite population can give the information on parasite diversity, population structure and the undergoing transmission intensity (Abdullah et al., 2013; Ferreira et al., 2007). Based on the Pvmsp-1 genetic marker, this study has found 13 allelic variants in segment F1, F2, and F3 of this gene. Although the number of samples was very low, the results revealed a low allelic variation among both population with mostly mono genotype of F1, F2, or F3 and multiple genotypes of F1 and F2 was only found in one isolate. The findings confirm the low endemic status of the studied districts as evidenced by the low API of Central Kalimantan Province, the ages of patients, which were only adult patients. A positive correlation between the rate of polyclonal infections and endemicity of malaria endemic provinces has been observed in parasite populations from Indonesia, where variation in the polyclonal *P. vivax* infection across sites was highly significant in contrast with that of *P. falciparum*, which was not significantly different (Noviyanti et al., 2015).

Multiple genotypes reveal a multiclonal infection can be the result of several bites of infected mosquitoes, or a single mosquito bite which is containing sporozoites that genetically are different (Conway et al., 1999). Genetic recombination of sexual stages in a mosquito host is continually occurred in natural population, which enable the rapid evolution of new strains, and resulting in multiple strains of the parasite which transmitted simultaneously, producing multiclonal infection in human host (Walliker et al., 1971; Conway et al., 1999). Multiclonal infection in *P. vivax* also can be caused by reactivation of dormant-hypnozoite during blood stage infection (Getachew et al., 2015). Multiple clone activation of hypnozoite can cause relapse in *P. vivax* infection, which resulted from either homologous or heterologous hypnozoite or both (de Araujo et al., 2012).

The low allelic variation might reveal the low transmission of malaria. Population genetic studies have demonstrated that areas with higher malaria transmission tend to have higher genetic diversity (Nkhoma et al., 2012). The problem is that there is no easy way to directly translate parasite genetic diversity into a level of parasite transmission (Greenhouse et al., 2015). The small number of samples in this study might not represent the entire studied area, but at least it has provided the genetic information of malaria based on the allelic diversity of the Pvmsp-1 gene which has been recorded as basic data of molecular epidemiology in the studied area.

Limitation of this study is mainly attributed to the quantity and quality of archived specimens used for DNA analysis. Nevertheless, owing to the impaired health surveillance and service due to pandemics, the current tool may provide further confirmation and/or validation to the results of blood film microscopy reading and RDT.

## Conclusion

The low allelic variation of Pvmsp-1 gene observed in this study may reflect the actual situation of the low malaria endemic status of the study sites. Further, systematic archived samples analysis in different areas of the districts and Province may better reflect the ongoing malarial situation.

### Conflicts of Interest

The authors declare that there is no conflict of interest regarding this study.

**List of Abbreviations:**
Pvmsp-1: *Plasmodium vivax* merozoite surface protein-1
PCR: Polymerization Chain Reaction
bp: base pair
API: Annual Parasite Incidence
GSBS: Giemsa-Stained Blood Smears
RDT: Rapid Diagnostic Test
PCR-RFLP: Polymerase Chain Reaction and Restriction Fragment Length Polymorphism
MOI: Multiplicity of Infection.
